# Emergence of 5-HT5A signaling in parvalbumin neurons mediates delayed antidepressant action

**DOI:** 10.1038/s41380-019-0379-3

**Published:** 2019-02-25

**Authors:** Yotam Sagi, Lucian Medrihan, Katia George, Miles Barney, Kathryn A. McCabe, Paul Greengard

**Affiliations:** Laboratory of Molecular and Cellular Neuroscience, The Rockefeller University, New York, NY USA

**Keywords:** Neuroscience, Depression

## Abstract

The behavioral response to antidepressants is closely associated with physiological changes in the function of neurons in the hippocampal dentate gyrus (DG). Parvalbumin interneurons are a major class of GABAergic neurons, essential for DG function, and are involved in the pathophysiology of several neuropsychiatric disorders. However, little is known about the role(s) of these neurons in major depressive disorder or in mediating the delayed behavioral response to antidepressants. Here we show, in mice, that hippocampal parvalbumin interneurons express functionally silent serotonin 5A receptors, which translocate to the cell membrane and become active upon chronic, but not acute, treatment with a selective serotonin reuptake inhibitor (SSRI). Activation of these serotonergic receptors in these neurons initiates a signaling cascade through which Gi-protein reduces cAMP levels and attenuates protein kinase A and protein phosphatase 2A activities. This results in increased phosphorylation and inhibition of Kv3.1β channels, and thereby reduces the firing of the parvalbumin neurons. Through the loss of this signaling pathway in these neurons, conditional deletion of the serotonin 5A receptor leads to the loss of the physiological and behavioral responses to chronic antidepressants.

## Introduction

The most commonly used antidepressant drugs, the selective serotonin reuptake inhibitors (SSRIs), have been shown to act in part by promoting a series of synaptic, cellular and network adaptations in the dentate gyrus (DG) [1–4]. Granule cells, the main neuronal type in the DG, are essential for the chronic response to SSRIs through 5-HT1A receptors [4], but their activity occurs within a dynamic neuronal microcircuitry under the control of interneuron populations of the DG. Two different classes of serotonin receptors, 5-HT1B and 5-HT2A, located on cholecystokinin (CCK)-expressing inhibitory interneurons, are critical for both the acute and chronic responses to SSRIs [5]. Their immediate activation by serotonin (5-HT) changes the activity of both granule cells and parvalbumin (PV) neurons and initiates the antidepressant response [5]. The activation of PV interneurons promotes the survival and development of newborn granule cells [6], and this process is associated with chronic SSRI treatment [3], but whether PV neurons are directly regulated by 5-HT has not been determined.

## Materials and methods

### Cell culture

80% confluent N2A cells (0.5 × 10^6^ cells/well) were transfected with a plasmid expressing the mouse 5-HT5A gene (4 μg DNA/well). Next day, 5-HT was pre-incubated for 30 min and forskolin was co-incubated for 15 min. Protein concentration was determined using BCA (Thermo Fisher Scientific, Waltham, MA). cAMP was determined by immunoassay kit (Assay Designs, Ann Arbor, MI). Baseline cAMP levels were 7.47 ± 1.34 pmol/mg protein.

For the determination of Ser 503 pKv phosphorylation level by Western blot, HEK293 cells (4 × 10^5^ cells/well) were transiently transfected with a plasmid expressing the mouse Kv3.1β (2 μg DNA/well). The following day, cells were pre-incubated with okadaic acid (20 nM final concentration) for 15 min. Phorbol 12-myristate 13-acetate (pma, 100 nM) and forskolin (50 μM) were added for 5 min. The cells were washed twice with PBS and immediately lysed in lysis buffer with protease and phosphatase inhibitor cocktails. 20 μg protein was loaded for Western Blot analysis. Kv3.1β and phospho-Kv3.1β were detected using mouse anti- Kv3.1β (UC DAVIS, 75–041), and rabbit anti- Ser-503 Kv3.1β (Phosphosolutions, p1550-503).

### Animals

All procedures were approved by the Animal Care and Use Committees of the Rockefeller University. Animals were maintained on a C57/Bl6N background and were housed in a 12-hour light/ dark interval with food and water ad-libitum. CCK^*TRAP*^, PV^*TRAP*^ and GAD^*TRAP*^ mouse lines were generated by crossing mice expressing loxP-stop-loxP-EGFP-RPL10a sequence in the Eef1α1 promoter (EEF1A1–LSL.EGFPL10) [7] with the respective (CCK^tm1.1(cre)Zjh/J^), (Pvalb^tm1(cre)Arbr/J^) and (GAD2^tm2(cre)Zjh/J^) *Cre* lines. TRAP qPCR analyses including mRNA isolation, cDNA amplification, qPCR analysis of mRNA levels were as previously described [5]. 5-HT5A floxed mice were generated by introducing loxP sites in the promoter and exon 1 of *htr5a*. Animals were crossed with PV^*Cre*^ (Pvalb^tm1(cre)Arbr^/J) mice. The modified gene sequences were determined using Southern blot. The functionality of the floxed mice was validated by breeding them with Nestin^*Cre*^ mice (B6.Cg(SJL)-TN (Nes^*Cre*^)1Kln). The resulting loss of mRNA expression of *htr5a* in the Cre positive but not in the negative offspring was confirmed using semi-quantitative qPCR. Females and males were used for immunohistochemical and biochemical studies and males for behavioral studies.

### Protein level of 5-HT5AR

The two hippocampi were pooled from each freshly dissected mouse and the membrane fraction was isolated using the Mem Per Plus kit (Thermo Fisher Scientific, Waltham, MA). Protein concentration was determined using the BCA protein assay (Thermo Fisher Scientific, Waltham, MA). 20 μg protein was loaded on 4–12% Bis-TRIS gels and were transferred to a PVDF membrane. Proteins were detected using antibodies for 5-HT5A (LifeSpan Biosciences, Seattle, WA), β-Actin and PSD-95 (both from Cell Signaling).

### Immunohistochemistry

Staining and analysis of 5-HT5A and Ser503 phospho Kv3.1β (pKv3.1β) were done for all samples at the same time using commercial antibodies against 5-HT5A (LifeSpan Biosciences, LS-A2119), Ser503 phospho Kv3.1β (Phosphosolutions, p1550-503), and PV (Sigma Aldrich, P0388). For detection, secondary Alexa goat anti- mouse or goat anti-rabbit were used (Thermo Fisher Scientific, Waltham, MA). Auto fluorescence was used to detect mCherry and cell nuclei were detected using DRAQ5 (Thermo Fisher Scientific, Waltham, MA). 4–6 coronal sections of 80 μm thickness were stained per antibody per mouse. To quantify the number of PV cells co-expressing 5-HT5A and pKv3.1β, the pixel mean value of 5-HT5A or pKv3.1β inside a PV cell was divided by its level outside the cell, using a custom written Matlab code [5]. A total of 24.2 ± 3.79 PV+ cells of the sub-granular zone of the ventral dentate gyrus were used per mouse. Cells with 5-HT5A, pKv3.1β mean pixel values above 140% of the background were considered immunopositive.

### Behavioral assays

Behavioral tests were conducted by a researcher unaware of the genotype, as previously described [5]. Chronic treatment consisted of 18 days of free drinking of fluoxetine (0.167 mg/ml)/saccharine (1%) mixture or saccharine alone (as vehicle). Acute fluoxetine (7.5 mg/kg) or saline (as vehicle) was administered intraperitoneally, 15 min before the test. For chemogenetic studies, one dose of clozapine N-oxide (4 mg/kg in saline, Sigma Aldrich, C0832) was injected intraperitoneally 30 min before the test.

### Electrophysiology

Whole-cell patch-clamp recordings from PV neurons were performed as described in the Extended Data Supplementary Methods section.

### Statistical analysis

Unless mentioned otherwise, all data are expressed as means ± s.e.m. Sample size was chosen based on previous reports to ensure adequate power. Statistical analysis was performed using GraphPad Prism 5/8.0.1. In all experiments, *P* < 0.05 was considered significant.

## Results

**Fig. 1:**
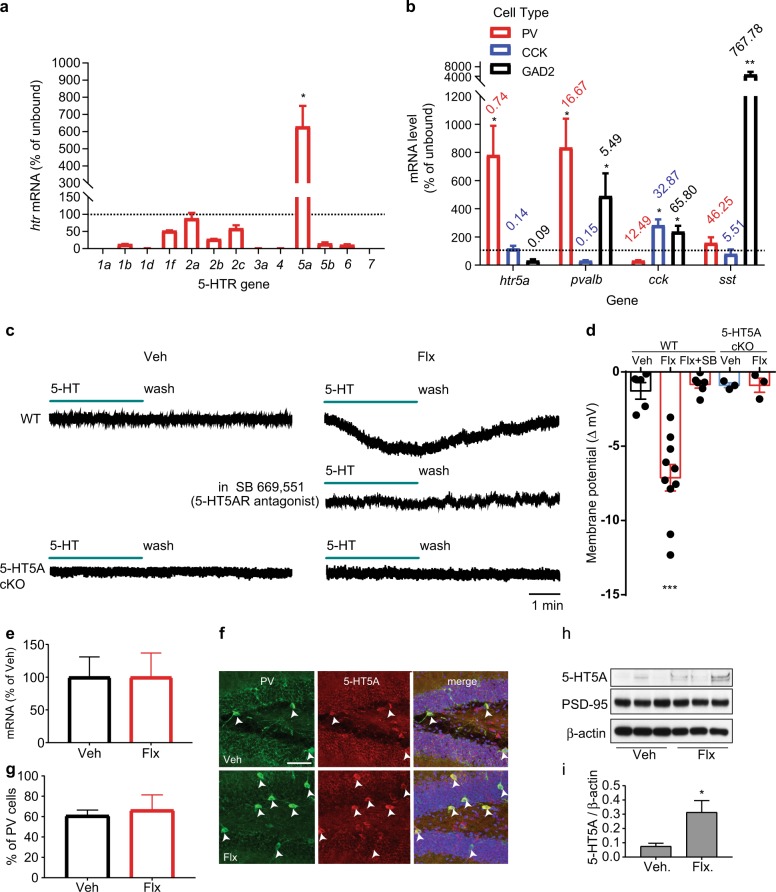
5-HT5A mediates inhibition in PV DG neurons after fluoxetine. **a** 5-HT receptor mRNA levels in hippocampal PV cells relative to the unbound input (*n* = 3 pairs each pulled from 3 mice). Bars represent means + SD. Paired *t*-test, *P < 0.05. Dashed line represents enrichment threshold. **b** mRNA levels of four genes in hippocampal PV-, CCK- and GAD2-expressing cells (*n* = 3, 3 per group). Bars represent means + SD. Numbers represent mRNA level as percent of *gpadh*. Paired *t*-test, *P < 0.05, **P < 0.01 vs. unbound. **c** Representative traces from PV DG neurons in WT and 5-HT5A cKO mice. **d** Membrane potential changes by 5-HT in Flx-treated (*n* = 10 neurons, 6 mice), and vehicle-treated (*n* = 5, 3) WT mice, or in slices preincubated with SB-669,551 (10 µM) (*n* = 6, 3). 5-HT had no effect on membrane potential in either Veh-treated (*n* = 3, 2) or Flx-treated (*n* = 3, 2) cKO mice. One-way ANOVA with post hoc Bonferroni tests, F (4, 22) = 6.29, ***P < 0.001. **e** TRAP analysis of 5-HT5A mRNA level in hippocampal PV cells after treatment with vehicle (*n* = 3, 9) or fluoxetine (*n* = 3, 9). Bars represent means as percent of Veh + SD. Unpaired *t*-test. **f** Representative images of 5-HT5A immunolabeling in SGZ PV cells from mice treated with vehicle or fluoxetine. Arrowheads indicate PV + cells; scale bar, 50 µm. **g** Quantitation of the percentage of SGZ PV cells co-expressing 5-HT5A. Bars represent mean percent from Veh (194 cells from 3 mice) or Flx (131, 3) + SD. Unpaired *t*-test. **h** Representative western blot image of 5-HT5A, PSD-95 and β-actin protein levels in the membrane-bound fractions of hippocampal lysates. **i** The 5-HT5A/β-actin ratio in the membrane bound fraction (*n* = 6 mice per treatment). Unpaired *t*-test, *P < 0.05

To investigate the presence of 5-HT receptors on hippocampal PV neurons, we used a translating ribosome affinity purification (TRAP) approach and generated PV-TRAP conditional knock-in mice. 5-HT5A receptors are enriched in hippocampal PV neurons (Fig. 1a), but not in CCK- or in other GAD65-expressing cells (Fig. 1b). This distribution of serotonin receptors is true only for the 5-HT5A receptor. Remarkably, using whole-cell patch-clamp recordings in acute hippocampal slices from DG PV neurons, acute application of 5-HT (1–100 µM) in basal conditions did not produce any change in the membrane potential (Fig. 1c, d; Extended Data Fig. 1a, b). However, after 3 weeks of chronic treatment with the SSRI fluoxetine (Flx; 0.167 mg/ml in drinking water), application of 5-HT led to a hyperpolarizing response of the membrane potential of PV neurons (Fig. 1c, d). This response to 5-HT was blocked by the 5-HT5A receptor antagonist SB 699,551 and was abolished in mice in which the 5-HT5A receptor was conditionally deleted from PV neurons (cKO) both before and after the antidepressant treatment (Fig. 1c, d). Fluoxetine treatment had no effect on either 5-HT5A receptor mRNA or protein immunolabeling in PV neurons (Fig. 1e–g), but resulted in an increase in 5-HT5A level at the membrane fraction of the hippocampal lysate (Fig. 1h, i), suggesting a possible translocation of the receptor to the plasma membrane in response to chronic fluoxetine.

**Fig. 2:**
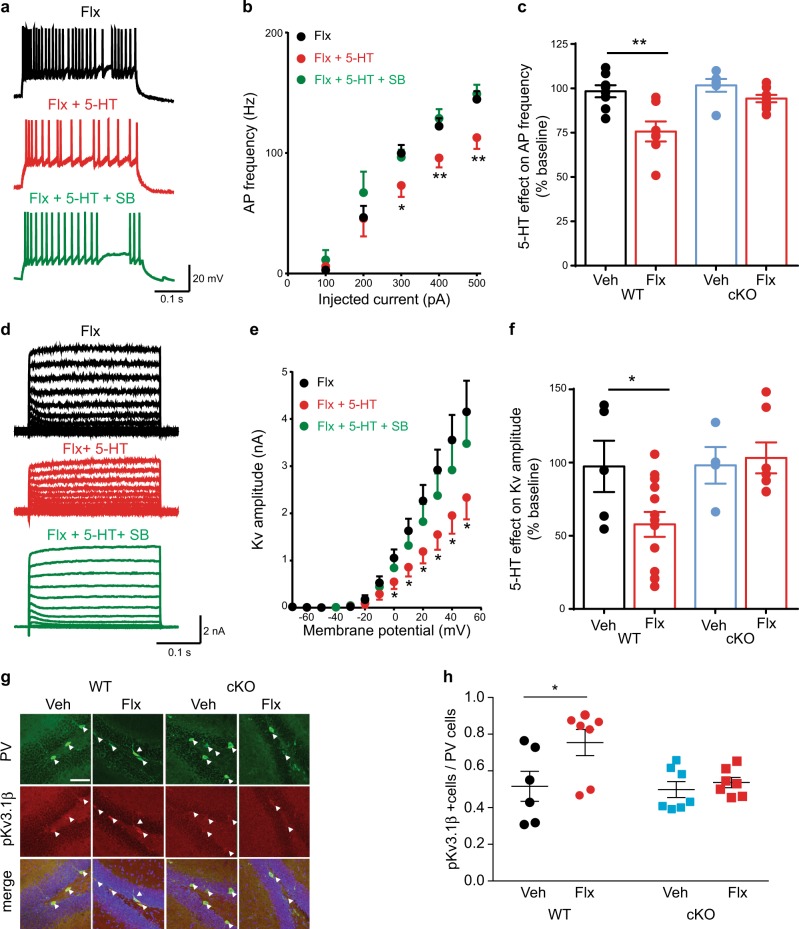
5-HT5AR mediates inhibition in Kv current after chronic fluoxetine. **a** Action potential (AP) firing in DG PV neurons in Flx-treated WT mice in response to 400 pA injected current. **b** *f*–*I* plot showing that 5-HT decreases AP frequency in Flx-treated WT mice, an effect rescued by the consecutive application of SB-669,551 (10 µM) (*n* = 7, 5). RM one-way ANOVA, *P < 0.05, **P < 0.01. **c** 5-HT decreased the frequency of APs in Flx-treated WT mice (*n* = 7, 5), but not in Veh-treated WT (*n* = 8, 6) or in Veh-(*n* = 6, 4) or Flx-treated (*n* = 9, 6) cKO mice. Two-way ANOVA, *F* genotype X treatment [1,22] = 7.67, **P < 0.01. Each dot represents a neuron. **d** Representative traces of Kv potassium currents in PV neurons from Flx-treated WT mice. The currents were evoked with 10 mV potential steps from −70 to +50 mV before and after 5-HT or 5-HT with SB-669,551 (10 µM). **e** *I*–*V* plot showing that 5-HT decreased the amplitude of Kv currents in Flx-treated WT mice, an effect partially rescued by SB-669,551 (10 µM) (*n* = 8, 4). RM One-way ANOVA, *P < 0.05. **f** 5-HT decreased the amplitude of the Kv current in Flx-treated WT mice (*n* = 13, 6), but not in Veh-treated WT (*n* = 5, 3), Veh- treated cKO (*n* = 4, 3) or Flx-treated cKO mice (*n* = 7, 7). Two-way ANOVA, *F* genotype X treatment [1,25] = 4.32, *P < 0.05. Each dot represents a neuron. **g** Representative images of pKv immunolabeling in SGZ PV cells from WT and cKO mice treated with Veh or Flx. Arrowheads indicate PV+ cells; scale bar, 50 µm. **h** Dot plot analysis of the percentage of SGZ PV cells coexpressing pKv (Veh:147 cells from 6 WT mice and 169 cells from 7 cKO mice; Flx:170 cells from 7 WT mice and 194 cells from 7 cKO mice). Two-way ANOVA, *F* Drug [1,23] = 5.30, *P* = 0.03, *P < 0.05 by post hoc Bonferroni

We next examined the effect of 5-HT, acting through 5-HT5A receptors, on the physiology of DG PV neurons by using whole-cell patch-clamp recordings in acute hippocampal slices (Fig. 2; Extended Data Fig. 1c–h). We observed that application of 5-HT decreases the firing frequency of these cells in wild-type (WT) mice after fluoxetine treatment, but not in vehicle-treated WT mice or in the cKO mice (Fig. 2a–c and Extended Data Fig. 2a). Furthermore, this effect was partially reversed by blocking the 5-HT5A receptors with SB 699,551 (Fig. 2a, b and Extended Data Fig. 2a), whereas 5-HT2A receptors did not influence the firing of these neurons (Extended Data Fig. 2b). It was shown previously that 5-HT5A acts through G-protein-coupled inwardly rectifying potassium channels (GIRKs) [8, 9]. However, application of 5-HT (30 µM) had no effect on the amplitude of the GIRK current in DG PV neurons from either vehicle- or fluoxetine-treated WT or cKO mice (Extended Data Fig. 3a).

Since the excitability of PV neurons is highly modulated by Kv3 potassium channels which are highly specific to and abundant in these neurons [10], we next investigated the effect of 5-HT on the amplitude of the Kv current before and after fluoxetine treatment. Application of 5-HT reduced the amplitude of Kv currents by almost 50 % in DG PV neurons from fluoxetine-treated WT mice, with no effect on vehicle-treated WT or cKO mice (Fig. 2d–f and Extended Data Fig. 3b), suggesting that the effect of 5-HT on the firing rate of PV neurons regulates the activity of Kv channels through 5-HT5A receptors.

It has been reported that the phosphorylation of Kv3.1β at serine 503 by protein kinase C (PKC) leads to a reduction in neuronal firing [11]. To study whether chronic SSRIs could modulate the Kv current by regulating the phosphorylation level of the channel, we next quantified the levels of pKv3.1β in the DG PV cells of vehicle- and fluoxetine-treated WT and cKO mice and observed a significant increase in the fraction of PV cells expressing pKv3.1β in WT mice after treatment with fluoxetine (Fig. 2g, h). Furthermore, this increase in the expression of the phosphorylated form of Kv3.1β correlated with the time-course for the appearance of the effects of 5-HT on the firing frequency of the PV neurons following fluoxetine treatment (Extended Data Fig. 4).

**Fig. 3:**
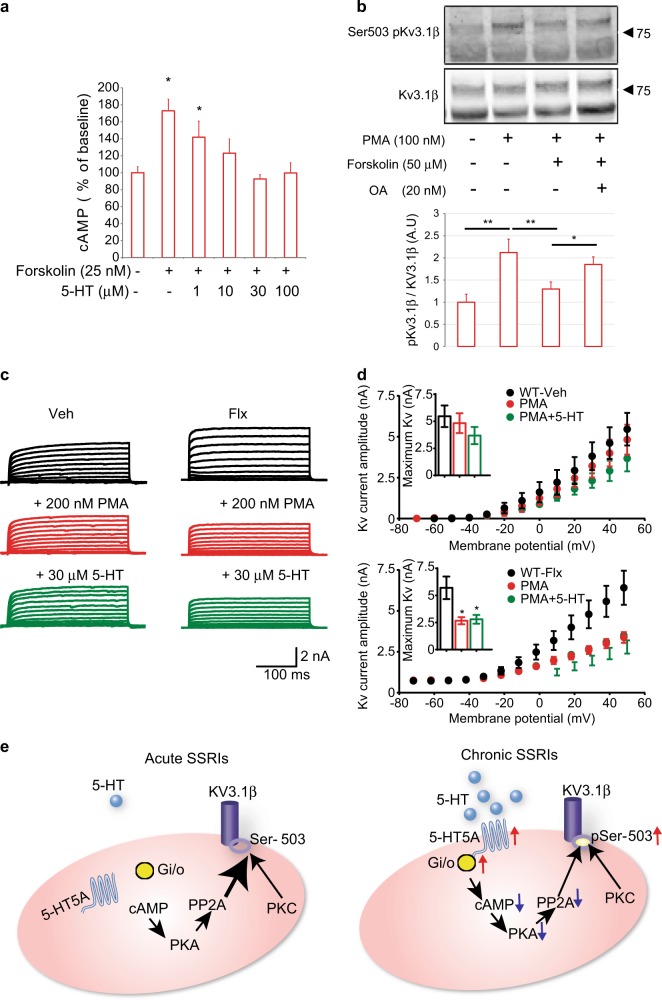
5-HT5A signaling in PV neurons. **a** Bar graph summary of cAMP level in N2A cells transiently transfected with mouse 5-HT5A. The cells were incubated with the indicated amounts of forskolin and 5-HT. One representative experiment out of three is shown. Bars represent means of cAMP level (*n* = 4 wells per group) as percentage of baseline + SD. One Way ANOVA, *P < 0.05 vs. baseline. **b** Representative western blot images (top) and bar graph summary (bottom) depicting the levels of Kv3.1β and Ser503 pKv3.1β in transfected HEK293 cells treated as indicated (*n* = 4 wells per condition). Bars represent mean densities of the pKv normalized to that of the total Kv. Number and arrowhead indicate protein size in kDa. One-way ANOVA. *P < 0.05, **P < 0.01. OA okadaic acid, PMA Phorbol 12-myristate 13-acetate. **c** Representative traces of Kv potassium currents evoked with 10 mV potential steps from −70 to +50 mV in PV DG neurons from Veh- and Flx-treated WT mice. **d** PMA (200 nM) decreased the amplitude of KV currents in Flx-treated mice (*n* = 4, 3) but not Veh-treated WT mice (*n* = 3, 3). RM one-way ANOVA, *P < 0.05. **e** Model for serotonergic signaling in PV hippocampal cells. Left: initial exposure to SSRIs does not alter cAMP levels and PKA activity. PP2A activity remains high and Kv3.1β is dephosphorylated. Right: chronic SSRIs increases 5-HT5A surface levels. 5-HT5AR activation reduces cAMP levels and diminishes PKA and PP2A activities to reduce Ser 503 Kv3.1β dephosphorylation

5-HT5A receptor was shown to have multiple mechanisms including coupling with Gi/Go proteins and its activation could reduce intracellular cAMP levels [12]. To measure the influence of 5-HT5A on cAMP levels, we used an in vitro approach and treated N2A cells previously transfected with 5-HT5A receptors with different concentrations of 5-HT. The increase in cAMP levels induced by the addition of forskolin was gradually reduced by increasing concentrations of 5-HT (Fig. 3a), suggesting that activation of 5-HT5A might inhibit the activity of Protein Kinase A (PKA). We next tested how changes in PKA activity might influence the phosphorylation of Kv channel. It has been reported that the phosphorylation of Kv3.1β channels is inhibited by protein phosphatase 2A (PP2A) [13]. Since activation of PKA increases PP2A activity [14], we next tested the idea that cAMP signaling could attenuate the phosphorylation of Kv3.1β. Indeed, in HEK293 cells, treatment with the adenylyl cyclase activator forskolin reduced the amount of the phosphorylated Kv3.1β (pKv3.1β) that was induced by Phorbol 12-myristate 13-acetate (PMA), a PKC activator, and this reduction was reversed by the addition of the PP2A inhibitor, okadaic acid (Fig. 3b), indicating that PKA and PKC act in antagonism on the phosphorylation and function of Kv3.1β channels. Moreover, in whole-cell patch-clamp experiments, the addition of PMA decreased the amplitude of the Kv current in DG PV neurons from fluoxetine-treated WT mice, with 5-HT having no further effects (Fig. 3c, d), further supporting the idea that over the course of chronic SSRI treatment, activation of the membrane bound- Gi-coupled 5-HT5AR would lower cAMP levels and disinhibit the phosphorylation of Kv3.1β at serine 503 (Fig. 3e).

**Fig. 4:**
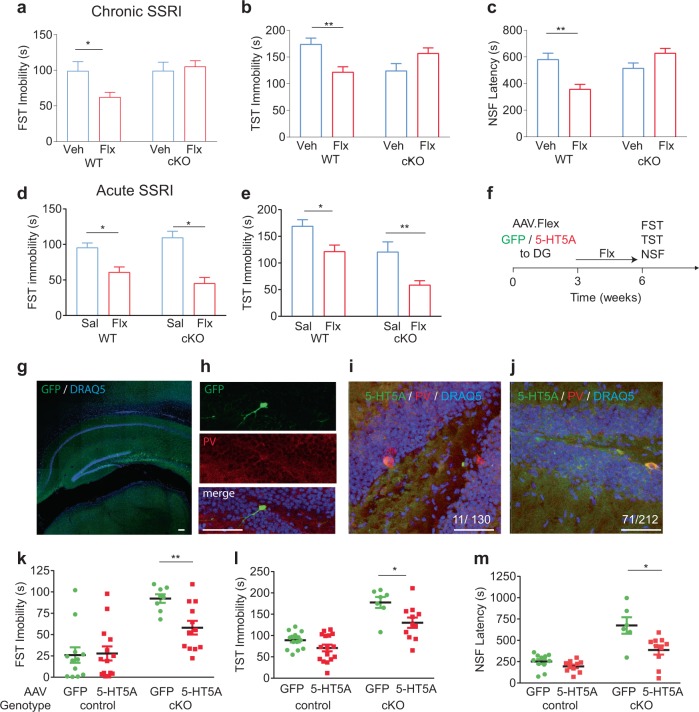
5-HT5AR mediates behavioral response to chronic SSRIs. **a** Immobility time in the forced swim test (FST) after drinking saccharine alone (Veh) or fluoxetine/saccharine mixture (Flx) for 18 days in WT or 5-HT5A cKO. Two-way ANOVA, *F* [1,44] = 4.58, *P* = 0.038. **b** Immobility time in the tail suspension test (TST) after Veh or Flx (for 18 days) in WT or cKO. Two-way ANOVA, *F* [1,42] = 13.83, *P* = 0.006. **c** Latency to approach food pellet in the novelty suppressed feeding (NSF) after Veh or Flx (for 18 days) in WT or 5- cKO. Two-way ANOVA, *F* [1,41] = 18.58, *P* < 0.0001. **d** Immobility time in FST 15 min after a single saline (Sal) or fluoxetine (Flx) intraperitoneal injection in WT or cKO. Two-way ANOVA, *F* genotype X treatment [1,34] = 3.99, *P* = 0.07; *F* treatment [1,34] = 37.27, *P* < 0.0001. **e** Immobility time in TST 15 min after a single injection of Sal or Flx in WT or cKO. Two-way ANOVA, *F* genotype X treatment [1,24] = 0.30, *P* = 0.590; *F* treatment [1,24] = 17.47, *P* = 0.0003; *F* genotype [1,24] = 17.93, *P* = 0.0003. *P < 0.05, **P < 0.01 ***P < 0.001 by post hoc Bonferroni. **f** Study design of viral mediated 5-HT5A gene delivery to PV-Cre and 5-HT5A cKO mice. **g** GFP-positive cells in the SGZ and hilus but not in the CA1 or outside the hippocampus after AAV.Flex-GFP injection to the DG. Scale bar: 100 μm. **h** GFP in a SGZ PV+ neuron. Scale bar: 50 μm. **i**, **j** Colocalization of 5-HT5A in PV neurons after injection of AAV.Flex-GFP (**i**) or AAV.Flex-5-HT5A (**j**) to 5-HT5A cKO. Numbers represent the fraction of SGZ PV+ cells immunopositive for 5-HT5A. Scale bars: 50 μm. **k** Immobility time in the FST after 18-day fluoxetine treatment in PV-Cre (control) injected with AAV.Flex-GFP or AAV.Flex-5-HT5A or in cKO. Two-way ANOVA, *F* genotype X AAV [1,42] = 4.54, *P* = 0.039. **l** Immobility time in TST. Two-way ANOVA, *F* genotype X AAV [1,44] = 2.37, *P* = 0.138; *F* genotype [1,44] = 60.62, *P* < 0.0001; *F* AAV [1,44] = 12, *P* = 0.0012. **m** Latency in NSF. Two-way ANOVA, *F* genotype X AAV [1,33] = 5.75, *P* = 0.022. *P < 0.05, **P < 0.01 by post hoc multiple *t*-tests with FDR

We next studied the role of the 5-HT5A receptors in PV neurons in regulating the behavioral response to SSRIs. Chronic SSRI treatment markedly reduced the immobility of WT mice in the forced swim test (FST) but failed to show an effect in the cKO mice (Fig. 4a). In the tail suspension test (TST), chronic fluoxetine treatment decreased the immobility of WT mice but did not induce any behavioral improvement in the cKO mice (Fig. 4b). In the novelty suppressed feeding test (NSF), a test associated with the beneficial effects of antidepressants in the DG [3], chronic fluoxetine treatment reduced the latency to approach the food pellet by WT mice, but not that by the cKOs (Fig. 4c). On the contrary, a single acute injection of fluoxetine produced a similar behavioral response in both WT and cKO mice (Fig. 4d, e), supporting the idea that 5-HT5ARs are essential only for the chronic antidepressant response. The daily consumption of fluoxetine and the amount of the food pellet consumed in the NSF test were not different in 5-HT5A cKO relative to that in WT mice, ruling out metabolic deficits in these mice (Extended Fig. 5).

To confirm the role of 5-HT5AR in DG PV neurons in mediating the behavioral response to chronic SSRIs, we used viral mediated gene delivery system to induced the levels of 5-HT5A in DG PV cells in control animals as well as to restore its expression in these cells from cKO mice. To this end, PV Cre control mice or 5-HT5A cKO mice were stereotaxically injected with AAV-Flex expressing either GFP or 5-HT5A constructs to the DG (Fig. 4f). Immunostaining experiments confirmed the spatial and temporal specificity of the expression of these transgenes (Fig. 4g–j). Importantly, restoring 5-HT5A level in PV cells in cKO mice rescued the antidepressant response to chronic fluoxetine treatment (Fig. 4k–m), whereas induction of 5-HT5AR in DG PV cells of WT mice did not alter the effect by the SSRI (Fig. 4k–m), supporting the idea that the functionality of this receptor in PV cells of the DG is essential in mediating the delayed antidepressant response to chronic SSRIs.

**Fig. 5:**
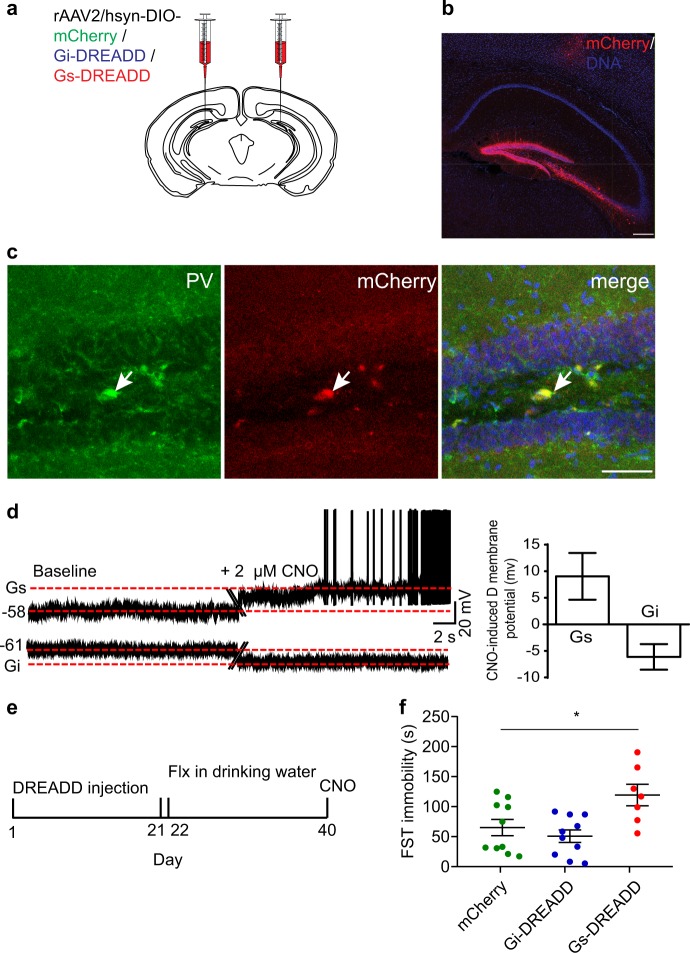
Activating cAMP signaling in PV DG cells attenuates the response to chronic fluoxetine. **a** Chemogenetic manipulation of PV DG cells consisted of bilateral injection of 1 μl rAAV2/hsyn-DIO- expressing one of three constructs: Gs-DREADD, Gi-DREADD or mCherry. **b** Immunofluorescence image depicting the expression of mCherry following Gs-DREADD delivery. Scale bar: 200 μm. **c** Immunolabeling confirmed that all mCherry immunopositive cells co-expressed PV. Scale bar: 50 μm. **d** Representative traces of patch-clamp recordings in hippocampal slices from mice in which PV neurons were infected with Gs-DREADD or Gi-DREADD. The right panel shows the hyperpolarization/depolarization of the membrane potential induced by the application of 2 µM CNO on the PV neurons infected with Gi-DREADD (−6.13 ± 2.4 mV *n* = 4, 3) or Gs-DREADD (9.03 ± 4.4, *n* = 3, 3). **e** Fluoxetine was administered for 18 days in the drinking water after which a single dose of CNO (4 mg/kg, intraperitoneally) was injected 30 min before the test. **f** Immobility time in the FST in PV-Cre mice injected with mCherry (*n* = 10 mice), Gi-DREADD (*n* = 10 mice) or Gs-DREADD (*n* = 6 mice). One-way ANOVA *F* [2,24] = 6.22, *P* = 0.007. *P = 0.023 by post hoc Dunnet

To study how changes in cAMP signaling in PV cells might affect the behavioral response to chronic antidepressant treatments, we used a chemogenetic approach and infected PV DG cells with rAAV2/hsyn-DIO- viruses with either hM4D (Gi)-mCherry or rM3D (Gs)-mCherry DREADDs (Designer Receptors Exclusively Activated by Designer Drugs), or with one that only expresses mCherry, as control (Fig. 5a). Immunolabeling study conducted 21 days after a bilateral delivery of the Gs-DREADD virus to the DG confirmed the specific expression of the mCherry protein in PV cells throughout the vDG (Fig. 5b). Visual inspection identified co-expression of PV in 84/84 of mCherry immunopositive cells (Fig. 5c). Patch clamp recordings confirmed the functionality of the DREADD receptors in PV cells, and showed depolarization or hyperpolarization of the membrane potential of PV cells infected with either Gs- or Gi-DREADDs respectively, in response to clozapine N-oxide (CNO, 2 µM), the ligand of the DREADD receptors (Fig. 5d). To test the effect of PV cell activation on the behavioral responsivity to SSRIs, mice were subjected to one injection of CNO (4 mg/kg, intraperitoneally) 30 min before the FST following 18-days of fluoxetine (Fig. 5e). Chronic antidepressant treatment resulted in reduced immobility in mCherry- and Gi-DREADD-treated mice. In contrast, CNO increased the immobility in Gs-DREADD injected mice (Fig. 5f), supporting the idea that the inhibition of PV cells is required for the responsivity to chronic SSRIs.

## Discussion

Here, we describe a role for 5-HT5AR in mediating the delayed antidepressant action of SSRIs by modulating the activity of PV hippocampal neurons. 5-HT5A receptors are expressed exclusively in the brain, mainly in the cerebral cortex, hippocampus and cerebellum [15, 16]. Although this 5-HTR isoform was associated with psychiatric disorders such as schizophrenia and unipolar depression [15, 17], research on its role has been hindered by the lack of specific agonists. On the basis of our findings, 5-HT5A receptor-selective ligands might show utility in the treatment of depression.

The only ion channel previously reported to be regulated by 5-HT5A was a GIRK rectifier [8, 9]. However, here we found that, in DG PV cells, 5-HT5A modulates the function of Kv3.1β. A similar mechanism was previously shown for other 5-HTRs, including 5-HT1B and 5-HT2A receptors that inhibit Kv channels in smooth muscle, partly by mediating changes in the phosphorylation of the channel [18]. The lack of effect by 5-HT on GIRK conductance in PV cells suggests that the signaling pathway for this receptor is cell-type-specific. Moreover, in contrast to the immediate activity of 5-HT5A in regulating cAMP level in transfected cells as well as on GIRK in cortical cells, the activity of 5-HT5A in DG PV cells requires chronic SSRIs treatment, indicating that both the coupling of 5-HT5A as well as the regulation of its surface level are cell-type and circuit-specific [9].

Several 5-HT receptor isoforms in DG cells have been implicated in the behavioral response to chronic SSRIs in different neuronal populations, but the behavioral response to chronic SSRIs coincides with an increase in the activity of 5-HTRs only in PV cells [4, 5, 19]. In addition to the identification of 5-HT5A in PV cells, we previously showed that 5-HT1B, 5-HT2A and 5-HT2C are enriched in CCK cells and we characterized the role of 5-HT2A in mediating the chronic response to SSRIs by inhibiting CCK cells in the DG. In contrast to the delayed onset of 5-HT5A signaling in PV cells, 5-HT2A signaling in CCK cells regulated the function of GIRK channels and mediated hyperpolarization in CCK cells in response to 5-HT exposure in mice that are naïve to SSRIs. Further, the response by 5-HT2A in CCK cells was abolished after chronic SSRI treatment, and this loss of function was mediated by transcriptional downregulation of the 5-HT2A gene. In contrast, our current study found that the expression level of 5-HT2A and 5-HT2C is not enriched in PV cells, suggesting that these receptors are not present in PV-expressing cells. Furthermore, chronic SSRI did not up-regulate the mRNA level of any 5-HTR in PV cells. Our TRAP and immunocytochemistry data support the idea that 5-HT5A is the only 5-HTR expressed in PV cells. Electrophysiological experiments showed that this receptor is initially silent and becomes functional only after chronic SSRI treatment. Furthermore, the lack of modulation of Kv current by PKC in PV cells from vehicle-treated mice supports the idea that the PKC signaling that regulates this channel in PV cells is activated only after chronic SSRIs.

In line with the idea that reducing cAMP signaling in PV cells by 5-HT5A is required for the delayed response to SSRIs, the activation of cAMP signaling in PV neurons by Gs-DREADD attenuated the behavioral response to the SSRI. Interestingly, inhibiting PV cells by Gi-DREADD did not improve the immobility relative to that in the mCherry-treated mice, suggesting that the endogenous 5-HT5A signaling is sufficient in attenuating cAMP signaling in these cells after chronic SSRI treatment. This is in line with the fact that the viral-mediated upregulation of 5-HT5A in WT mice did not improve the response to SSRI relative to that by the control virus.

While 5-HT signaling in PV-expressing DG cells is required for the response to chronic SSRIs, 5-HT signaling in CCK expressing DG cells mediates both the initial as well as the delayed responses to these drugs [5]. This is mediated by the activation of 5-HT1B and 5-HT2A receptors, which synergistically inhibit the neurotransmission from CCK cells as well as their activity [5]. Importantly, the delayed activation of the 5-HT5A receptors in PV cells after chronic SSRI treatment coincides with the transcriptional down-regulation of the 5-HT1B and 5-HT2A receptors in CCK cells [5]. Taken together, these data support a revised monoaminergic hypothesis in which SSRIs achieve their antidepressant response by acting on different receptors and cell types in a temporally, rather than spatially, orchestrated fashion.

## Supplementary information

Extended Information

## References

[1] Hu H, Gan J, Jonas P (2014). Interneurons. Fast-spiking, parvalbumin(+) GABAergic interneurons: from cellular design to microcircuit function. Science.

[2] Marin O (2012). Interneuron dysfunction in psychiatric disorders. Nat Rev Neurosci.

[3] Santarelli L, Saxe M, Gross C, Surget A, Battaglia F, Dulawa S (2003). Requirement of hippocampal neurogenesis for the behavioral effects of antidepressants. Science.

[4] Samuels BA, Anacker C, Hu A, Levinstein MR, Pickenhagen A, Tsetsenis T (2015). 5-HT1A receptors on mature dentate gyrus granule cells are critical for the antidepressant response. Nat Neurosci.

[5] Medrihan L, Sagi Y, Inde Z, Krupa O, Daniels C, Peyrache A (2017). Initiation of behavioral response to antidepressants by cholecystokinin neurons of the dentate gyrus. Neuron.

[6] Song J, Sun J, Moss J, Wen Z, Sun GJ, Hsu D (2013). Parvalbumin interneurons mediate neuronal circuitry-neurogenesis coupling in the adult hippocampus. Nat Neurosci.

[7] Stanley S, Domingos AI, Kelly L, Garfield A, Damanpour S, Heisler L (2013). Profiling of glucose-sensing neurons reveals that GHRH neurons are activated by hypoglycemia. Cell Metab.

[8] Grailhe R, Grabtree GW, Hen R (2001). Human 5-HT(5) receptors: the 5-HT(5A) receptor is functional but the 5-HT(5B) receptor was lost during mammalian evolution. Eur J Pharmacol.

[9] Goodfellow NM, Bailey CD, Lambe EK (2012). The native serotonin 5-HT(5A) receptor: electrophysiological characterization in rodent cortex and 5-HT(1A)-mediated compensatory plasticity in the knock-out mouse. J Neurosci.

[10] Rudy B, McBain CJ (2001). Kv3 channels: voltage-gated K+channels designed for high-frequency repetitive firing. Trends Neurosci.

[11] Song P, Kaczmarek LK (2006). Modulation of Kv3.1b potassium channel phosphorylation in auditory neurons by conventional and novel protein kinase C isozymes. J Biol Chem.

[12] McCorvy JD, Roth BL (2015). Structure and function of serotonin G protein-coupled receptors. Pharmacol Ther.

[13] Song P, Yang Y, Barnes-Davies M, Bhattacharjee A, Hamann M, Forsythe ID (2005). Acoustic environment determines phosphorylation state of the Kv3.1 potassium channel in auditory neurons. Nat Neurosci.

[14] Ahn JH, McAvoy T, Rakhilin SV, Nishi A, Greengard P, Nairn AC (2007). Protein kinase A activates protein phosphatase 2A by phosphorylation of the B56delta subunit. Proc Natl Acad Sci USA.

[15] Thomas DR (2006). 5-ht5A receptors as a therapeutic target. Pharmacol Ther.

[16] Pasqualetti M, Ori M, Nardi I, Castagna M, Cassano GB, Marazziti D (1998). Distribution of the 5-HT5A serotonin receptor mRNA in the human brain. Brain Res Mol Brain Res.

[17] Birkett JT, Arranz MJ, Munro J, Osbourn S, Kerwin RW, Collier DA (2000). Association analysis of the 5-HT5A gene in depression, psychosis and antipsychotic response. Neuroreport.

[18] Cogolludo A, Moreno L, Lodi F, Frazziano G, Cobeno L, Tamargo J (2006). Serotonin inhibits voltage-gated K+currents in pulmonary artery smooth muscle cells: role of 5-HT2A receptors, caveolin-1, and KV1.5 channel internalization. Circ Res.

[19] Schmidt EF, Warner-Schmidt JL, Otopalik BG, Pickett SB, Greengard P, Heintz N (2012). Identification of the cortical neurons that mediate antidepressant responses. Cell.

