# Supplementary material for: Emergence of 5-HT5A signaling in parvalbumin neurons mediates delayed antidepressant action
Source: Mol Psychiatry. 2019 Feb 25;25(6):1191–201. doi: 10.1038/s41380-019-0379-3 (PMC7244406; doi:10.1038/s41380-019-0379-3)
Supplement: Supplementary file 1 — Extended Information [file 41380_2019_379_MOESM1_ESM.pdf]

Extended Information Emergence of 5-HT5A Signaling in Parvalbumin Neurons Mediates Delayed Antidepressant Action. Sagi Y., Medrihan, L., George, K., Barney, M., McCabe, K. and Greengard P.

Extended Materials and Methods:

Electrophysiology:

Mice between 8 and 12 weeks of age were euthanized with CO_2. After decapitation and removal of the brains, transversal slices (400 µm thickness) were cut using a Vibratome 1000 Plus (Leica Microsystems, USA) at 2 °C in a NMDG-containing cutting solution (in mM): 105 NMDG (N-Methyl-D-glucamine), 105 HCl, 2.5 KCl, 1.2 NaH_2PO_4, 26 $NaHCO_3$, 25 Glucose, 10 $MgSO_4$, 0.5 $CaCl_2$, 5 L-Ascorbic Acid, 3 Sodium Pyruvate, 2 Thiourea (pH was around 7.4, with osmolarity of 295–305 mOsm). After cutting, slices were left to recover for 15 minutes in the same cutting solution at 35 °C and for 1 h at room temperature (RT) in recording solution (see below). Whole-cell patch-clamp recordings were performed with a Multiclamp 700B/Digidata1550A system (Molecular Devices, Sunnyvale CA, USA). EGFP-positive PV neurons were selected for recording based on the expression of the fluorescent marker using an upright Olympus BX51WI microscope equipped with the appropriate filters (Olympus, Japan) and a SPECTRA X LED light engine (Lumencor, OR, USA). The extracellular solution used for all recordings contained (in mM): 125 NaCl, 25 $NaHCO_3$, 2.5 KCl, 1.25 NaH_2PO_4, 2 $CaCl_2$, 1 $MgCl_2$ and 25 glucose (bubbled with 95% O_2 and 5% CO_2). The slice was placed in a recording chamber (RC-27L, Warner Instruments, USA) and constantly perfused with oxygenated aCSF at 24 °C (TC-324B, Warner Instruments, USA) at a rate of 1.5–2.0 ml/min.

For measuring 5-HT-induced responses, whole-cell current-clamp recordings were obtained from PV neurons using recording pipettes (King Precision Glass, Inc, Glass type 8250) pulled in a horizontal pipette puller (Narishige) to a resistance of 3–4 MΩ. The intracellular solution contained (in mM): 126 K-gluconate, 4 NaCl, 1 $MgSO_4$, 0.02 $CaCl_2$, 0.1 BAPTA, 15 glucose, 5 HEPES, 3 ATP, 0.1 GTP (pH 7.3). For measuring the membrane potential 30 seconds of recording were binned into 0.5 ms bins and fitted with a Gaussian. 5-HT (1-10-30-100 µM) and SB-669,551 (10 µM) were applied in the recording chamber using an automatic valve MPS-2 multichannel perfusion system (World Precision Instruments, USA). Tetradotoxin (TTX, 0.3 µM) was added in the bath to prevent indirect responses from other neurons in the slices.

For measuring the action potential firing we used whole-cell current-clamp recordings from PV neurons. The intracellular solution contained (in mM): 126 K-gluconate, 4 NaCl, 1 $MgSO_4$, 0.02

$CaCl_2$, 0.1 BAPTA, 15 glucose, 5 HEPES, 3 ATP, 0.1 GTP (pH 7.3). Small currents were injected to the cells to bring the membrane potential at -70 mV. Consecutive current steps of 100 pA were injected to induce depolarization. The frequency of action potentials was measured using the first two action potentials evoked by the respective injected current. Action potential properties were measured from the first action potential to avoid any confounding effects of adaptation.

For measuring the Kv we used whole-cell voltage-clamp recordings from PV neurons. The intracellular solution contained (in mM): 126 K-gluconate, 4 NaCl, 1 $MgSO_4$, 0.02 $CaCl_2$, 0.1 BAPTA, 15 glucose, 5 HEPES, 3 ATP, 0.1 GTP (pH 7.3). Voltage steps of 10 mv (from −70 mV to 50 mV; 1s, every 10 s) were used to determine current-voltage (I-V) relationships. A P/4 protocol was used to eliminate leak currents. The amplitude of the current was measured on the steady-state part of the response. Tetradotoxin (TTX, 0.3 μM) was added in the bath to block Na^+ currents.

For measuring the GIRK we used whole-cell voltage-clamp recordings from PV neurons. The intracellular solution contained (in mM): 126 K-gluconate, 4 NaCl, 1 $MgSO_4$, 0.02 $CaCl_2$, 0.1 BAPTA, 15 glucose, 5 HEPES, 3 ATP, 0.1 GTP (pH 7.3).Voltage steps of 10 mv (from −120 mV to -70 mV; 1s, every 10 s) were used to determine current-voltage (I-V) relationships. A P/4 protocol was used to eliminate leak currents. The amplitude of the current was measured on the steady-state part of the response. Tetradotoxin (TTX, 0.3 μM) was added in the bath to block Na^+ currents.

For measuring the Gi-DREADD and Gs-DREADD effect on the membrane potential, mCherry-positive PV neurons were selected for recording based on the expression of the fluorescent marker. CNO (2 μM) was added to the bath and the membrane potential was measured as described above.

Data were acquired at a sampling frequency of 100 kHz, filtered at 1 kHz and analyzed offline using pClamp10 software (Molecular Devices, Sunnyvale, CA, USA). All electrophysiological data are expressed as means ± SEM. Statistical analysis was performed using the Student's t-test, One Way Analysis of Variance with Bonferroni *post hoc* comparison unless stated otherwise, with the help of GraphPad Prism 5. In all experiments, $P < 0.05$ was considered significant.

Extended Results:

Ex. Fig. 1

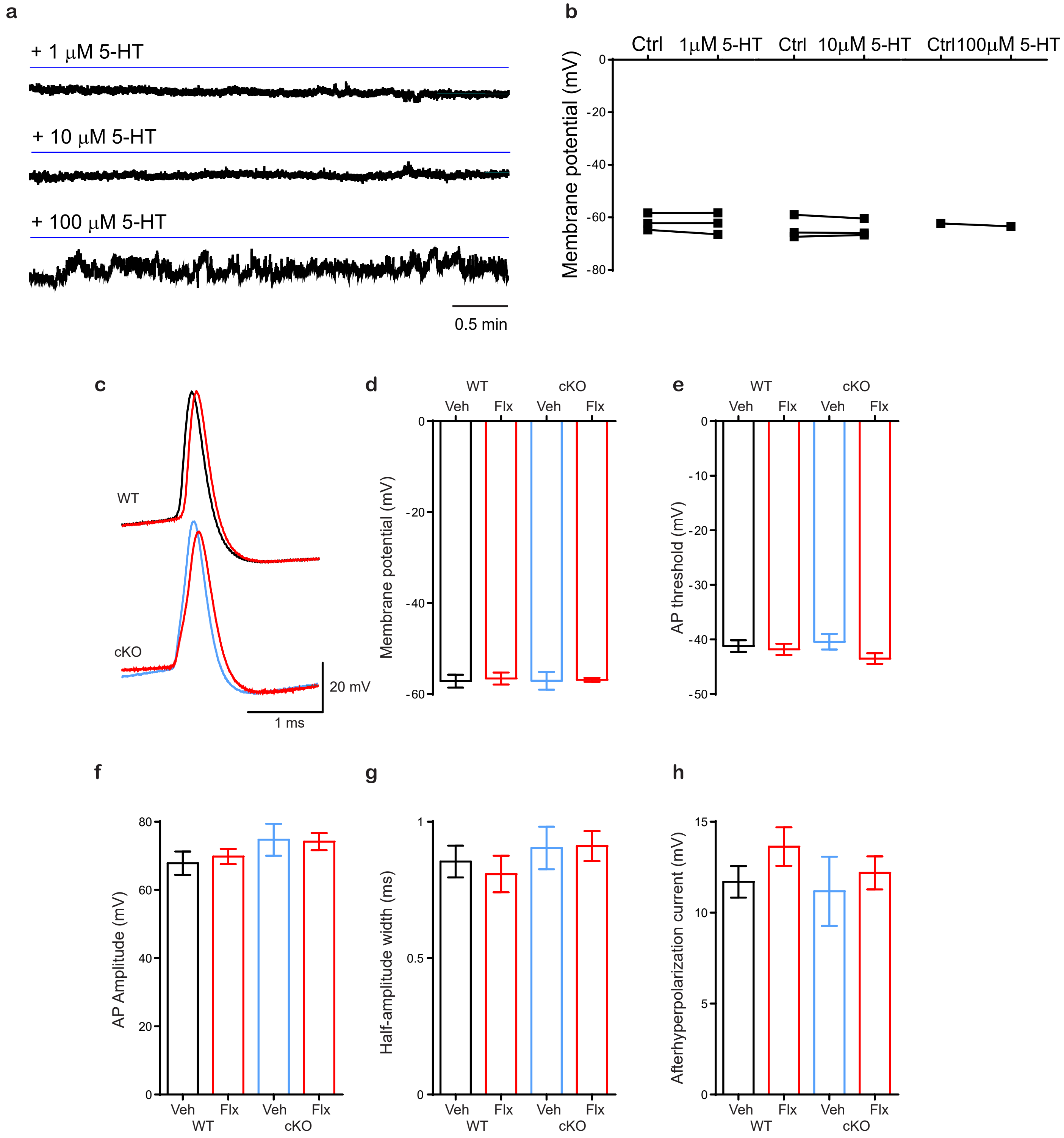
a
+ 1 μM 5-HT
+ 10 μM 5-HT
+ 100 μM 5-HT
0.5 min
b
Ctrl
1μM 5-HT
Ctrl
10μM 5-HT
Ctrl
100μM 5-HT
Membrane potential (mV)
0
-20
-40
-60
-80
c
WT
cKO
20 mV
1 ms
d
WT
cKO
Veh
Flx
Veh
Flx
Membrane potential (mV)
0
-20
-40
-60
e
WT
cKO
Veh
Flx
Veh
Flx
AP threshold (mV)
0
-10
-20
-30
-40
-50
f
AP Amplitude (mV)
80
60
40
20
0
Veh
Flx
Veh
Flx
WT
cKO
g
Half-amplitude width (ms)
1
0.5
0
Veh
Flx
Veh
Flx
WT
cKO
h
Afterhyperpolarization current (mV)
15
10
5
0
Veh
Flx
Veh
Flx
WT
cKO

Ex. Fig. 2

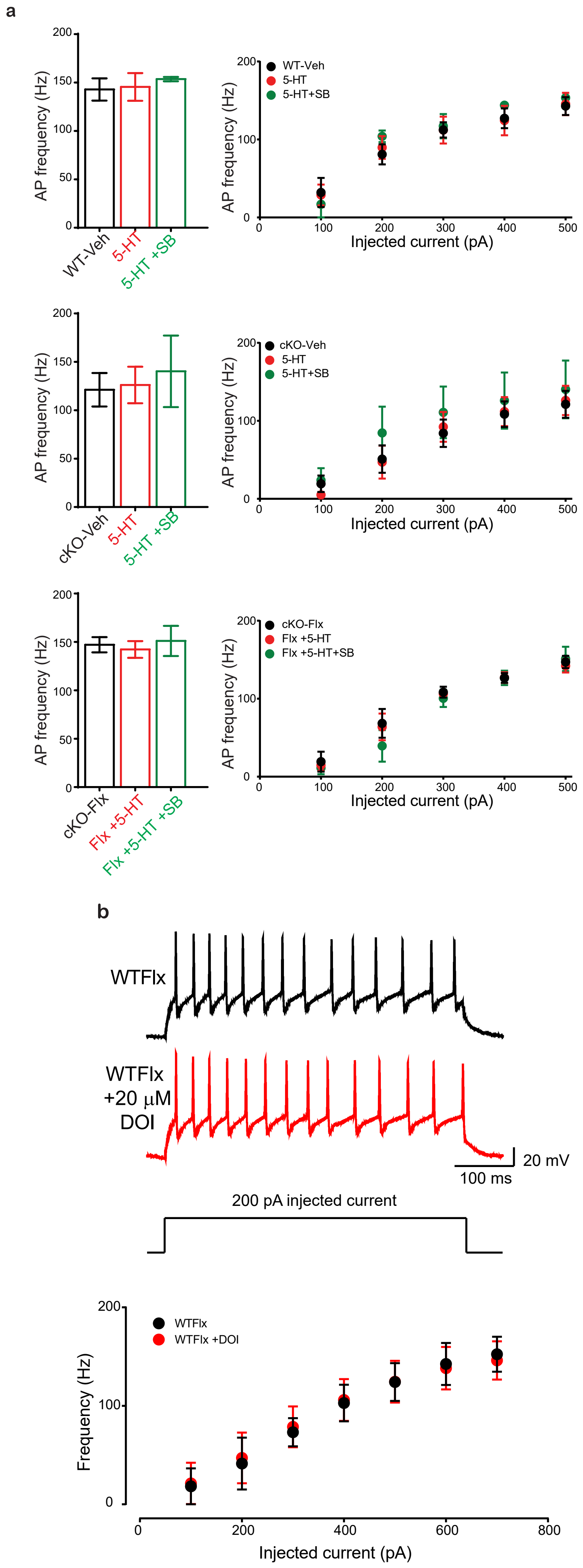
a
AP frequency (Hz)
WT-Veh
5-HT
5-HT +SB
5-HT+SB
Injected current (pA)
cKO-Veh
cKO-Flx
Flx +5-HT
Flx +5-HT +SB
Flx +5-HT+SB
b
WTFlx
WTFlx +20 μM DOI
20 mV
100 ms
200 pA injected current
Frequency (Hz)
WTFlx +DOI

Ex. Fig. 3

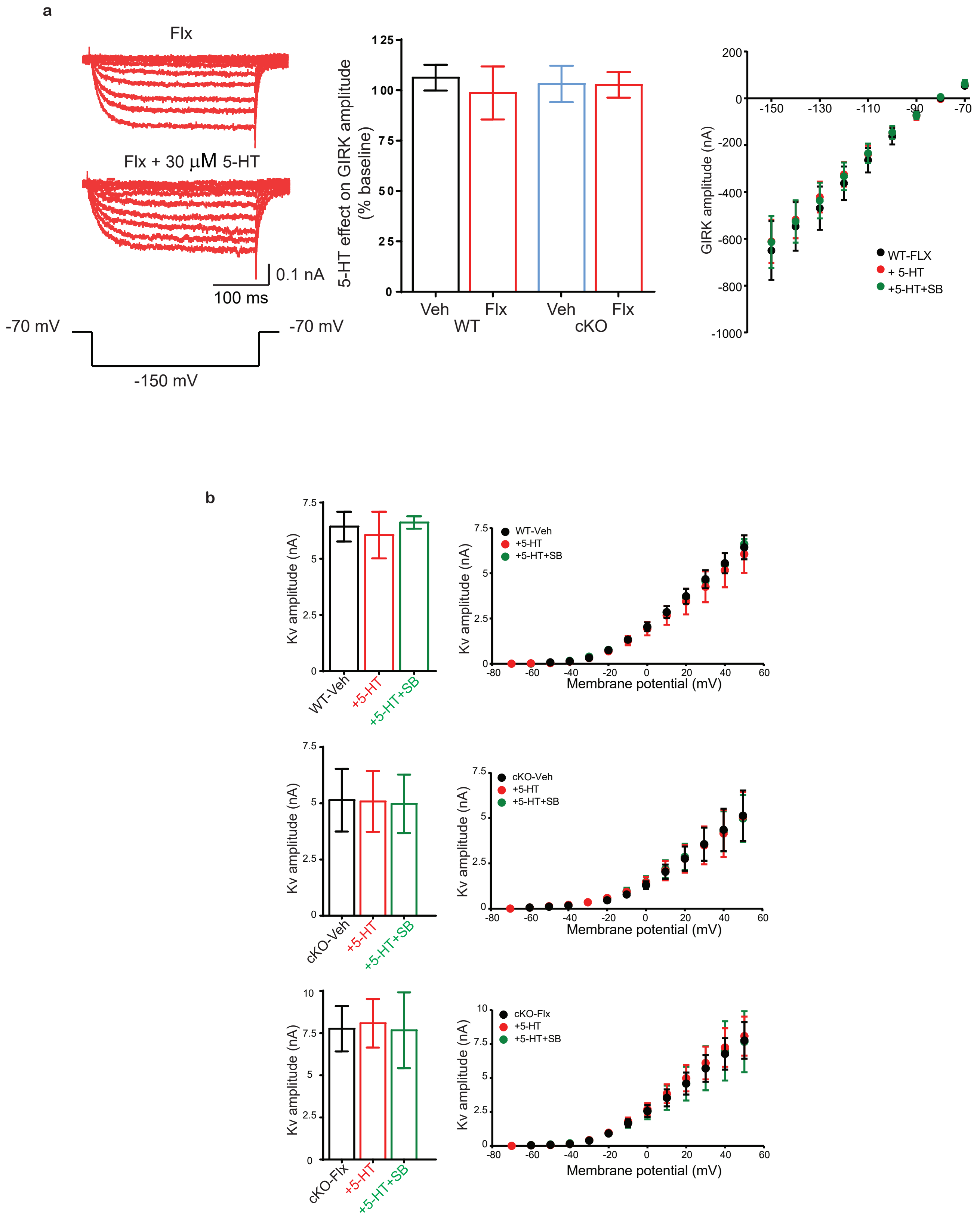
a
Flx
Flx + 30 μM 5-HT
0.1 nA
100 ms
-70 mV
-70 mV
-150 mV
5-HT effect on GIRK amplitude (% baseline)
Veh
Flx
Veh
Flx
WT
cKO
GIRK amplitude (nA)
WT-FLX
+ 5-HT
+5-HT+SB
b
Kv amplitude (nA)
WT-Veh
+5-HT
+5-HT+SB
Membrane potential (mV)
cKO-Veh
cKO-Flx

Ex. Fig. 4

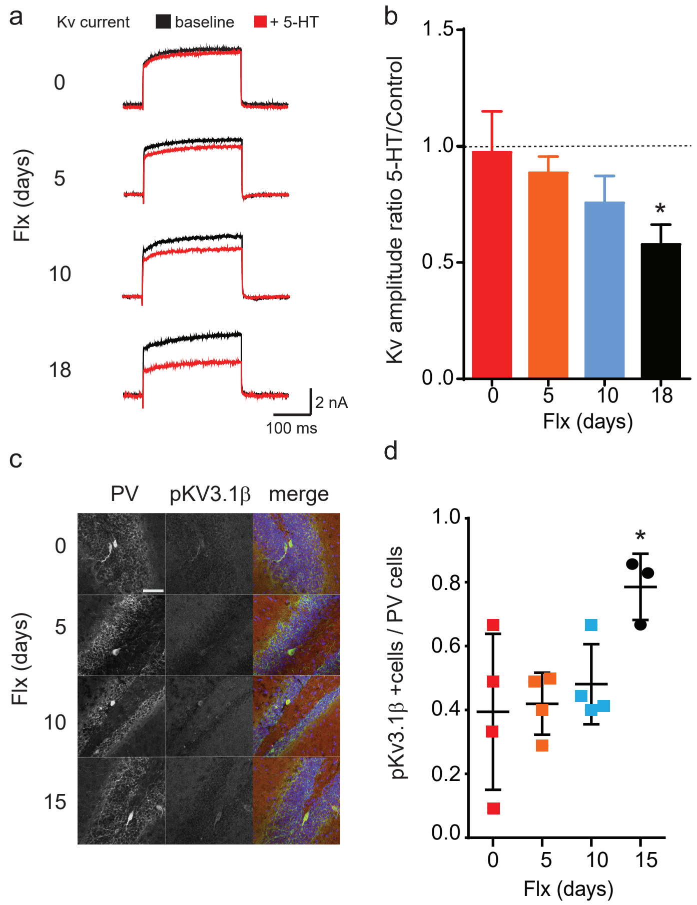

a
Kv current
baseline
+ 5-HT
Flx (days)
0
5
10
18
2 nA
100 ms
b
Kv amplitude ratio 5-HT/Control
1.5
1.0
0.5
0.0
*
0
5
10
18
Flx (days)
c
PV
pKV3.1β
merge
Flx (days)
0
5
10
15
d
pKv3.1β +cells / PV cells
1.0
0.8
0.6
0.4
0.2
0.0
*
0
5
10
15
Flx (days)

Ex. Fig. 5

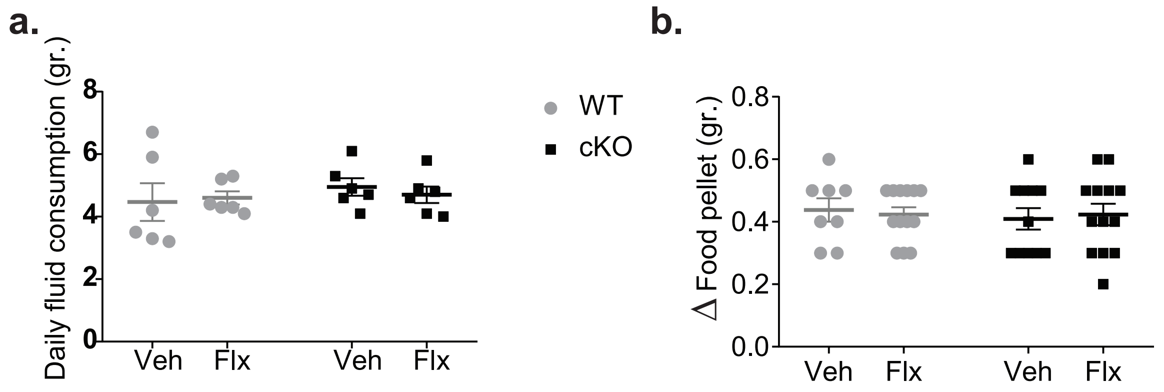
a.
Daily fluid consumption (gr.)
8
6
4
2
0
Veh
Flx
Veh
Flx
WT
cKO
b.
Δ Food pellet (gr.)
0.8
0.6
0.4
0.2
0.0
Veh
Flx
Veh
Flx

Legend to extended data.

Extended Figure 1. **a-b.** Representative traces of whole-cell current-clamp recordings showing the absence of any effect induced by different concentrations of 5-HT on the membrane potential of DG PV neurons in acute slices of WT mice and the respective before-after plots (**b**). **c**. Traces showing action potentials in control WT (black) and 5-HT5A conditional knock-out (blue) mice and in the same genotypes after chronic fluoxetine treatment (red). **d.** Histograms showing the mean ± sem membrane potential in WT and 5-HT5A conditional knock-out mice without or with chronic fluoxetine treatment. **e-h.** Histograms showing the mean ± sem of different action potential properties in WT and 5-HT5A conditional knock-out mice without or with chronic fluoxetine treatment. n = 14, 10 for naïve WT; n = 18, 11 for fluoxetine-treated WT; n = 8, 8 for naïve 5-HT5A cKO; n = 13, 8 for fluoxetine-treated 5-HT5A cKO.

Extended Figure 2. **a.** Absolute data showing no effect on firing frequency of DG PV neurons upon application of 5-HT and the consecutive application of the 5-HT5AR antagonist, SB 669,551 in vehicle-treated WT mice (top row), vehicle- treated cKO and fluoxetine-treated cKO mice (middle and bottom row, respectively). n = 6, 4 for naïve WT; n = 5, 3 for veh-treated 5-HT5A cKO; n = 5, 3 for fluoxetine-treated 5-HT5A cKO. **b.** Representative traces of action potential (AP) firing in DG PV neurons in Flx-treated WT mice in response to 400 pA injected current before (black) and after the addition of DOI hydrochloride (20 μM, red)**.** f-I plot showing that DOI hydrochloride (20 μM) has no effect on the firing of DG PV neurons in Flx-treated WT mice (n = 4, 3).

Extended Figure 3. **a**. Left: Representative traces of inward GIRK-mediated potassium currents evoked with 10 mV potential steps from -140 to -40 mV in fluoxetine-treated

WT mice in the absence or presence of 5-HT (30 μM). Middle: Bath application of 30 μM 5-HT had no effect on the maximum inward K^+ current in any of the tested genotypes. n = 3, 3 for naïve WT; n = 6, 4 for fluoxetine-treated WT; n = 5, 4 for naïve 5-HT5A cKO; n = 9, 5 for fluoxetine-treated 5-HT5A cKO. Right: I-V plot showing the GIRK current in chronic fluoxetine treated WT mice in different experimental conditions. **b.** Absolute data showing no effect on the amplitude of the Kv current in DG PV neurons upon application of 5-HT and the consecutive application of the 5-HT5AR antagonist, SB 669,551 in vehicle-treated WT mice (top row) and both vehicle- and fluoxetine-treated cKO mice (middle and bottom row, respectively). n = 9, 5 for naïve WT; n = 3, 3 for naïve 5-HT5A cKO; n = 5, 3 for fluoxetine-treated 5-HT5A cKO.

Extended Figure 4

a. Representative traces of Kv potassium currents evoked with a potential step from -70 to +50 mV in PV neurons from slices of WT mice at different time points of the fluoxetine treatment. **b**. Histograms showing the effect of the application of 30 μM 5-HT on the amplitude of the Kv current at the start and on different days of the Flx treatment in WT mice. Bars represent means ± sem. 0 days (5 neurons, 3 mice), 5 days (2, 2), 10 days (4, 2), and 18 days of Flx treatment (13, 6). (One-way ANOVA followed by *post hoc* Fisher's multiple comparison test, *$P < 0.05$). **c**. Representative images of Ser 503 pKv3.1β immunolabeling in SGZ PV cells from WT mice treated with Flx as indicated. scale bar, 50 μm. **d**. Dot plot analysis of the percentage of SGZ PV cells coexpressing pKv. Bars represent mean ratios from 0 days (4 mice and 101 cells, written as 4, 101), 5 days (4,103), 10 days (4, 107), and 15 days of Flx treatment (3, 70) ± sem. (One-way

ANOVA, *F* [3, 11] = 4.22, *P*= 0.03; followed by *post hoc* Tukey's multiple comparison test, **P*< 0.05).

Extended Figure 5.

a. Scatter dot plot summary of 24-hour long consumption of vehicle and fluoxetine in the drinking water by individual WT and 5-HT5A cKO mice (n= 6 mice per group). Two-way ANOVA, *F* genotype X treatment [1, 20] = 0.26, *F* genotype [1, 20] = 0.61, *F* treatment [1, 20] = 0.02. P> 0.05. **b**. Scatter dot plot summary of change in food pellet weight in the NSF test after vehicle and fluoxetine chronic treatment in WT (n= 8, 13) and 5-HT5A cKO mice (n= 11, 13). Two-way ANOVA, *F* genotype X treatment [1, 41] = 0.67, *F* genotype [1, 41] = 0.67, *F* treatment [1, 41] = 0.99. P> 0.05.
